# Psycho-emotional impact of the COVID-19 pandemic on nursing professionals in Ecuador: a cross-sectional study

**DOI:** 10.1186/s12912-024-02119-0

**Published:** 2024-07-03

**Authors:** Janet Vaca-Auz, Sonia Revelo-Villarreal, Jorge Luis Anaya-González, Cristina Vaca-Orellana, Rocío Castillo, Geovanna Altamirano-Zavala, Francisco Vicens-Blanes, Jesús Molina-Mula

**Affiliations:** 1 Universidad Técnica del Norte. Facultad de Ciencias de La Salud, Ibarra, Ecuador; 2 Nursing and Physiotherapy Department, University of Balearics Island, Ctra. Valldemossa km 7,5, Palma, 07122 Spain

**Keywords:** Anxiety, Depression, Post-traumatic stress, Burnout syndrome, COVID-19 pandemic, Ecuadorian nurses

## Abstract

**Aim and objectives:**

To analyse the levels of anxiety, depression, post-traumatic stress, and burnout among nursing professionals working in the Imbabura region of Ecuador during the COVID-19 pandemic and identify the contributing socio-occupational factors.

**Background:**

The high demand for care of COVID-19 patients led to increased work pressure on nurses, owing to increased demands for care and shortages of medical supplies and protective equipment.

**Design:**

A cross-sectional study was conducted from September to December 2022 using a self-administered questionnaire addressed to nursing professionals who cared for COVID-19 patients.

**Methods:**

The questionnaire included socio-demographic characteristics, the Spanish adaptation of Hospital Anxiety and Depression Scale (HADS-Spanish), Impact of Event Scale–Revised (IES-R) for the evaluation of post-traumatic stress disorder (PTSD), and the Spanish adaptation of the Maslach Burnout Inventory–Human Services Survey (MBI-HSS-Spanish) for burnout assessment. Univariate and multivariate analyses were performed.

**Results:**

Of the 782 participants, 88.6% had a high level of burnout (MBI-HSS-Spanish scale score > 27). Female nurses, nurses with eight-hour work shifts, and older professionals exhibited high levels of anxiety and depression. Prolonged working hours in COVID-19 patient care services were found to be a risk factor for burnout and post-traumatic stress.

**Conclusions:**

Participating nurses presented with a high level of chronic work stress and exhibited signs of anxiety and depression during the period under consideration. Providing nurses with psychological support measures and performing liaison consultations will alleviate the psychological burden on nurses.

**Relevance to clinical practice:**

The study has shown that accounting for the environments where the emotional impact is greatest and how to reduce it would not only reduce anxiety, depression, and burnout in nurses but also improve the quality of care, not only in pandemic.

**Patient or public contribution:**

Nurses contributed to the conduct of the study by participating in the data collection via questionaries.

**Supplementary Information:**

The online version contains supplementary material available at 10.1186/s12912-024-02119-0.

## What does this paper contribute to the wider global clinical community?

- Knowing which characteristics, environments, and processes are most damaging to nurses is critical to proactively address their emotional needs and prevent a breakdown of the healthcare system.

- Establishing strategies to address the emotional impact of daily practical activity is essential to improve the quality of life of nurses. It should not be a passive strategy but a proactive one to avoid situations like those experienced in the pandemic.

## Introduction

The COVID-19 pandemic has had serious consequences in all spheres of social development and has led to various magnitudes of inadequacies in public health services. The high demand for care of COVID-19 patients led to increased work pressure on nurses, among other health professionals, owing to increased demands for care, shortages of medical supplies and protective equipment, and other risks related to close and repeated contact with COVID-infected patients [1, 2]. Inordinate workload and prolonged stress to which these healthcare professionals were exposed during the pandemic adversely affectedheir work performance, psycho-emotional health, and overall quality of life [3].

In this context, several authors have noted that healthcare professionals frequently manifested anger, anxiety, apathy, and prolonged stresduring the pandemic [4]. These emotions were exhibited by changes in their behavioural patterns characterised by exhaustion, loss of motivation, and other symptoms that affected their work performance and put the safety of the patients themselves at risk, as highlighted by some authors [5].

Burnout syndrome is another aspect that was found to be aggravated by working conditions, unstable protocols, and reorganisation of the workspace [6].

From a social perspective, distancing measures, fear of contagion, and mourning the loss of friends and family, among other factors, have resulted in an increased prevalence of anxiety, depression, and post-traumatic stress disorder [7].

In the context of this study, it should be noted that in 2020, the Ecuadorian government declared a state of exception due to the rise in COVID-19 cases and exposure to a highly virulent strain originating from the United Kingdom [8]. In response to this emergency, which affected the public health of the Ecuadorian population, the National Emergency Operations Committee and all the institutions of the Risk System were activated, including all the health institutions in Zone 1 [8].

Previous studies in several countries, including France, Italy, and Spain, have highlighted the vulnerability of frontline health workers to the emotional impact of both the pandemic and its consequences [9].

The emotional and psychological toll of nurses' work had been recognized even before the onset of the COVID-19 pandemic. However, the pandemic and the subsequent surge in demand for healthcare services have placed an unprecedented strain on these frontline workers, exacerbating risk factors for their mental well-being. Increased direct personal interactions, heightened levels of responsibility, and frequent exposure to life-threatening situations have intensified the challenges faced by these professionals, amplifying the risks to their overall health.[10–12].

This dire situation compounds the findings of studies like that conducted by Fornés-Vives in 2019 [13], which had already projected that one in five nurses would experience significant work-related stress leading to attrition from the profession.

In a systematic review [14] on the prevalence of mental health disorders among healthcare workers during and after a pandemic, the authors concluded that the most prevalent psycho-emotional disorder observed among healthcare workers was PTSD (21.7%), followed by anxiety disorders (16.1%) and major depressive disorder (13.4%). The authors noted that age and level of staff exposure during care were critical modulating factors responsible for these disorders.

Another study conducted in the European region with a sample of 196 nurses reported the incidences of depression (16.8%), anxiety (46.4%), stress (22.4%), and burnout (50.5%) among these healthcare professionals. The scale scores showed that older professionals, those with the presence of comorbidities, fewer leisure activities, and increased working hours, were the most vulnerable [15].

In Latin American countries, nurses faced the COVID-19 pandemic with equipment and medical supplies shortag, work overload, understaffing, lack of infrastructure, and weakened healthcare systems, which may have led to severe emotional disturbances during the pandemic [16].

Several studies have demonstrated the important behavioural, affective, cognitive, and social impacts of the COVID-19 pandemic on professionals [17]. In this regard, the prevalence of the primary manifestations of psycho-emotional disorders is an alarming indicator of the need to manage behaviours appropriately and timely (19).

The above situation has been demonstrated to have led to multiple psychophysiological dysfunctions among healthcare professionals, which influence their family life as well as social and work environment. On the other hand, this differential exposure to risks contributes to the appearance of other problems such as burnout, post-traumatic stress, or depression as a consequence of the medium- and long-term effects of the COVID-19 infection [18].

A systematic review on the impact of the pandemic on the mental health of healthcare professionals concluded that work time, fear of becoming infected and infecting loved ones and/or patients, and concern about controlling the epidemic, among others, may be precipitating factors for an alteration in the mental health of healthcare professionals in times of a pandemic [19].

Like other countries, Ecuador has also been impacted by the above-mentioned consequences of the COVID-19 pandemic. According to data from the International Labour Organisation, in May 2021, the number of confirmed cases of COVID-19 registered was 324,482, while the number of COVID-related deaths that were registered was 16,738 [20]. As of March 2023, the figures are eight times more the above confirmed cases and three times more deaths, so the post-COVID impact has increased significantly [21].

A study conducted in several provinces of Ecuador during the pandemic found that 90% of nursing staff presented moderate-to-severe burnout syndromelobally and in the subscales of emotional exhaustion and depersonalisation [22].

In addition, other factors of exposure to critical life events such as prolonged social distancing, deaths of loved ones, low professional appraisal, and the performance of a multifunctional role are triggers of symptomatology in the psycho-biological sphere, such as avoidance, negative or guilty thoughts, irritability, insomnia, and difficulty in concentrating and managing feelings and emotions [23].

The results of this study will greatly contribute to understanding the events and situations that nurses face and how these factors impact their mental health allows for the establishment of improvement and prevention strategies. In this way, we can proactively mitigate an emotional collapse among nurses. Furthermore, these factors not only arise in pandemic situations but also regularly occur in daily practice. The pandemic may have subsided, but the effects on our nurses persist, and it is necessary to take action.

In this context, various factors such as work shifts (particularly rotating shifts including morning, afternoon, and night), increased monthly working hours, older age, and substantial professional experience (10–20 years in the same service) appear to be linked to a heightened risk of burnout among nurses. These findings underscore the necessity of documenting and monitoring the emotional, psychological, and social repercussions of burnout, alongside anxiety, depression, and posttraumatic stress, among nurses even prior to the pandemic [10].

Finally, a few risk factors for these emotional disorders have been identified for nurses, such as being female, having a lower socioeconomic status, and having a high risk of contracting COVID-19 as opposed to a few protective factors, such as sufficient healthcare resources, up-to-date and accurate information, and taking precautionary measures [4].

The aim of the study was to analyse the levels of anxiety, depression, PTSD, and burnout among nursing professionals working in Ecuador during the COVID-19 pandemic and the socio-occupational factors that may have contributed to their occurrence.

## Methodology

### Design

This cross-sectional analytical observational study conducted from 5 September to 30 December 2022 included 782 nursing professionals from first- and second-level healthcare facilities in in Zone 1 (territorial) of Ecuador.

### Participants

For operational planning, the Ecuadorian territory is divided into 9 zones. Zone 1 is located in the northern part of the country and includes three provinces: Esmeraldas province, situated in the coastal region, Imbabura in the highland region, and Carchi to the northeast.

Ecuador, one of the countries in the Andean region, stands out for its higher health expenditure relative to GDP. Nationally, there are 4,136 health establishments, with 621 offering inpatient services. [24] The healthcare system comprises two sectors: the public and the private. The former is supported by a social security system financed through contributions from formal sector workers, while the latter serves the population with higher purchasing power. The Ministry of Health, the Ecuadorian Social Security Institute (IESS), and NGOs are some of the institutions involved in the Ecuadorian healthcare system and operate independently.

Of the total professionals, 85.81% work in health establishments located in urban areas, while 14.19% perform their duties in rural areas. Nurses in rural areas are typically newly graduated professionals who fulfill rural service requirements. However, the total number of nurses in Ecuador still does not reach the recommended figures by the World Health Organization (WHO), which suggests having 25 to 30 nurses per 10,000 inhabitants. The nurse-to-population ratio in 2020 was 15.4 per 10,000 inhabitants nationwide, with an average rate in zone 1 of 15.7. The WHO recommends a ratio of 1:2 – 1:3 nursing professionals per population. In Ecuador, the current ratio is 1:12 in level I and II complexity institutions; however, it is even lower in level III facilities. Consequently, during the COVID-19 pandemic, nurses were compelled to extend their working hours from 12 to 24 continuous hours without relief shifts.[25].

A sample calculation was made, with a confidence of 95% and a precision of ± 5%, which considered a minimum size of 350 professionals and a replacement rate of 35% [26].

As this was a descriptive study, the maximum sample per population base was collected using a consecutive sampling to reach as many nurses as possible from the target population. Out of a total of 1,300 nurses working in Zone 1, information was collected from 782 professionals who agreed to participate in the study, representing 60.1% of potential study participants. Two percent of the questionnaires were discarded as they were incomplete.

### Inclusion and exclusion criteria

Nursing professionals who provided care to COVID-19 patients during the pandemic in health facilities of the first and second levels of care in < BLINDED FOR REVIEW > and agreed to participate in this study were included. Incomplete questionnaires were discarded.

#### Data collection (Procedures)

After the participants gave informed consent, their data were collected using an online or face-to-face questionnaire by consecutive responses on the socio-demographic and occupational characteristics of the professionals, factors related to COVID-19 care in healthcare facilities in Zone 1 of Ecuador and three validated scales: Spanish adaptations of the Maslach Burnout Inventory–Human Services Survey (MBI-HSS-Spanish) and Hospital Anxiety and Depression Scale (HADS-Spanish) and Impact of Event Scale–Revised (IES-R).

An ID and a link were assigned through the platform from September to December 2022. Dissemination mechanisms were used through local meetings with directors, process coordinators, and chief nurses of the selected health facilities in Zone 1.

### Instruments

The questionnaire was divided into four sections. The first section collected socio-demographic information (gender, age, marital status, and children) and information related to the working conditions in the respondent’s area of work (level of care, work shifts, work experience, experience in caring for covid patients), as well as some factors related to care during the pandemic.

The second section explored the burnout level through applying the MBI-HSS-Spanish scale. This instrument consists of 22 items and three dimensions: (a) emotional exhaustion, classified as ‘low’ for scores 0–18, ‘moderate’ for scores 19–26, and ‘high’ for scores above 27; (b) depersonalisation, considered ‘low’ for scores < 5, ‘moderate’ for scores 6–9, and ‘high’ for scores > 10; (c) sense of low personal achievement, perceived as ‘low’ for scores < 33, ‘moderate’ for scores 34–39, and high for scores > 40. Psychometric properties show adjustment in all three dimensions and consistency [27].

The third section consisted of HADS-Spanish [28]. This scale is composed of 14 interspersed items corresponding to the depression and anxiety subscales. The items are scored on a 4-point Likert scale, with cut-off values of < 7 for ‘absent’, 8–10 for ‘doubtful or possible’, and > 11 for ‘severe’. Anhedonia, that is the inability to derive pleasure from activities generally considered enjoyable, is included in this instrument as a key symptom to differentiate anxiety from depression. The psychometric properties of the scale confirm adequate internal consistency [29].

The fourth section included the items of the IES-R, whose purpose is to measure post-traumatic stress, understood as the emotional distress provoked by a life event, which is conceptualised as subjective stress. The scale consists of 22 items, included in three subscales: (a) intrusion, seven items; (b) avoidance (eight items), and (c) hyperarousal (seven items), on a scale of 0 to 4. The cut-off point of post-traumatic stress is 20 in this scale, and a score ≥ 20 suggests a possible psychiatric disorder, while a score 14–20 suggest that the presence of a disorder is unlikely [28]. The post-traumatic stress level corresponds to ‘sub-clinical’ for a score of 0–8. The post-traumatic stress level is considered correct from 100 to 200, 100–200, between 100 and 200 incorrect: from 100–200, between 100–200. According to several authors, the scale has adequate psychometric properties with a three-factor reliability of 0.70 in all subscales [30].

The psychometric quality of the Maslach Burnout Inventory scale is considered encouraging for its potential use in most studies on Burnout Syndrome in South America in its Spanish version. In Ecuador, in the study by Torres et al. [31], on Burnout Syndrome in Ecuadorian healthcare personnel during the COVID-19 pandemic, the Maslach Burnout Inventory questionnaire was used based on available scientific evidence and the specific needs of each study.

Regarding the use of the IES-R instrument to evaluate post-traumatic stress disorder, a study conducted in Ecuador in the context of the COVID-19 pandemic shows good results in terms of internal consistency of the instrument, with total IES-R coefficients (0.95, 0.91, and 0.95); intrusion/hyperactivation factor (0.95, 0.89, and 0.94); and avoidance factor (0.87, 0.84, and 0.84)[32] [33] Likewise, the HADS scale (the Spanish adaptation of Hospital Anxiety and Depression Scale) has been validated and used in various research studies in Ecuador, demonstrating its psychometric properties with a Cronbach's alpha of 0.86. [34].

#### Data analysis

Data were stored in Excel spreadsheets and then transferred to SPSS® Statistical Package for the Social Sciences (SPSS Inc., Chicago) version 27 for Windows. A *p*-value < 0.05 was considered statistically significant.

Prior to the analysis, reliability, normality, and homoscedasticity tests of the relevant variables were performed. Measures of location, distribution, central tendency, and dispersion were applied. The Kolmogorov–Smirnov test showed that the distribution of the data conformed to a normal distribution. Outliers or extreme values were treated.

A univariate descriptive analysis was performed, and the frequency distribution of each of the qualitative variables was determined. For quantitative variables, the mean and the standard deviation were used as measures of central tendency and dispersion, respectively.

The possible association between variables was explored. The chi-square test was used for the analysis of categorical variables. For the analysis between dichotomous categorical variables and quantitative variables, Student’s t-test was used for independent groups, with the requirement of normality and equality of variances. To explore the possible association between categorical variables with three or more levels and quantitative variables we used analysis of variance. The type of association between continuous quantitative variables was examined by determining Pearson’s correlation coefficient.

To study the relationship of the confounding factors (socio-demographic characteristics) with the result of obtaining the different measurements, a bivariate or multiple linear regression model was applied with those variables that reached a significance < 0.2 in the univariate comparisons, obtaining different risk estimates (odds ratios, ORs).

Linear regression equations were used to evaluate the relationship between each kind of variable (demographic, occupational, COVID-19, and burnout) regardless of the symptoms (post-traumatic stress, anxiety, and depression), using the R^2^ value (coefficient of determination) and the standardised coefficient. Finally, models were used to see which variables (demographic, occupational, COVID-19, and burnout) were jointly associated with symptoms of post-traumatic stress, anxiety, and depression. The model was estimated by least squares, using the forward extraction method.

### Validity and reliability/rigour

Scientifically validated scales were used to ensure adequate methodological rigour. The corresponding normality and homoscedasticity tests were applied, and intervals describing the confirmatory values were defined, where a confidence level of 95% was considered.

The findings of this study were compared with other similar studies. The possible limitations of this study have been addressed in end of this paper.

### Ethics approval and consent to participate

This study was evaluated and authorised by the Ethics Committee of the Faculty of Health Sciences of the Technical University of the North- Ecuador (nº012/2023). Confidentiality was respected by anonymising the data with alphanumeric codes, which were stored on a CD for the exclusive use of the researchers. Participants can request the cessation or cancellation of data at any time. The researchers declare that they have no conflict of interest.

The study respected the principles of bioethics when conducting health research. The procedures were conducted in accordance with the ethical considerations described in the Declaration of Helsinki and the Code of Good Research Practice. The clarifications about the study were transcribed in the Terms of Free and Informed Consent and made available to the participants. Completion of the instrument was considered as acceptance to participate in the research.

The informed consent of the participants was obtained after complete information about the objective of the study and the risks and benefits. The participant signed the informed consent form and agreed to participate in the study and then the data was obtained.

## Results

### Socio-demographic characteristics and information on the COVID-19 pandemic

The study population was predominantly female (*n* = 496, 63.42%). A large proportion of participants (*n* = 309, 39.51%) lied in an age range of 29–38 years. About half of the participants (*n* = 396, 50.6%) lived with a partner, and the majority had dependent children (*n* = 579, 74.04%). A small proportion of participants had postgraduate-level education (*n* = 109, 14%).

The 17.1% have between 10–15 years of work experience, followed by 16.2% with 4–6 years, 15.6% with 1–3 years, and 12.1% with 0–12 months. The 10.5% and 10.2% fall between 9–10 years and 7–8 years respectively. Only 8.8% and 2.7% are between 21–30 years and over 30 years respectively.

Staff with permanent contracts accounted for 30.5% (*n* = 235), and 45.5% (*n* = 356) worked rotating shifts (seven-hour morning or afternoon shifts and 10-h night shifts). The range of professional experience was 1–15 years, representing 47.4% (*n* = 371). The details are presented in Tables 1 and 2..

**Table 1:** Socio-demographic characteristics of participants

**Age (years)**	n	%
18–28	162	20.72%
29–38	309	39.51%
39–48	187	23.91%
49–58	108	13.81%
59–66	15	1.92%
> 66	1	0.13%
**Cohabitation**		
Companions	31	3.96%
Parents	234	29.92%
Couple	396	50.64%
Alone	121	15.47%
**Level of education**		
Bachelor’s degree	673	86.00%
Master’s Degree	109	14.00%
**Employment Status**		
Contract	235	30.05%
Temporary appointment	492	62.92%
Permanent appointment	55	7.03%

**Table 2:** Relationship of the scales to the study variables

Variable	EMOTIONAL FATIGUE				ANXIETY				DEPRESSION				POST-TRAUMATIC STRESS				DESPERSONALITATION				PERSONAL FULFILMENT			
	N	Mean	SD	*p* value	N	Mean	SD	*p* value	N	Mean	SD	*p* value	N	Mean	SD	*p* value	N	Mean	SD	*p* value	N	Mean	SD	*p* value
EXPERIENCE IN COVID- UNITS																								
0 - 2 months	183	20.81	11.704	0.251	183	9.05	2.660	0.47	183	11.02	2.760	0.001	183	24.04	13.640	0.373	183	5.63	5.600	0.042	183	29.32	10.748	0,000
3 - 4 months	83	20.96	10.867		83	8.71	2.860		83	10.94	3.070		83	24.83	12.970		83	5.63	5.690		83	31.12	10.695	
5 - 7 months	54	17.48	8.393		54	8.87	1.910		54	12.48	1.960		54	23.87	11.460		54	4.35	4.288		54	34.37	7.522	
8 - 10 months	64	20.84	12.100		64	9.42	2.450		64	11.66	2.460		64	27.86	15.230		64	5.14	5.258		64	28.48	10.464	
more than 10 months	398	19.71	10.229		398	8.95	2.190		398	11.57	2.280		398	24.68	13.160		398	4.33	5.345		398	33.27	8.260	
Total	782	20.04	10.719		782	8.98	2.390		782	11.45	2.510		782	24.75	13.33		782	4.84	5.394		782	31.80	9.498	
Type of health center																								
Hospitals	658	19.72	10.507	0.058	658	8.95	2.32	0.456	658	11.52	2.413	0.060	658	24.54	13.324	0.314	658	4.78	5.292	0.514	658	32.14	9.278	0.022
Primary care	124	21.71	11.681		124	9.13	2.714		124	11.06	2.986		124	25.85	13.379		124	5.13	5.922		124	30.01	10.449	
Total	782	20.04	10.71		782	8.92	2.39		782	11.45	2.516		782	24.75	13.333		782	4.84	5.394		782	31.8	9.498	
UNITS/ SERVICES																								
Direct care	28	15.79	7.857	0.322	28	9.11	1.641	0.314	28	11.07	1.942	0.123	28	26.32	12.41	0.836	28	3.14	3.263	0.396	28	33.18	8.079	0.153
Emergency	163	19.79	11.010		163	8.86	2.598		163	11.11	2.596		163	24.67	13.480		163	5.74	5.873		163	30.15	10.386	
Surgery Hospitalitation	57	20.32	7.863		57	8.93	2.178		57	11.68	2.399		57	24.11	12.280		57	4.65	5.201		57	32.40	8.571	
Gynecology Hospitalitation	69	19.30	10.694		69	8.88	2.111		69	12.07	2.205		69	23.51	13.125		69	4.67	5.011		69	30.97	8.911	
Ped Hospitalitation	24	20.83	11.064		24	9.17	2.371		24	11.96	2.404		24	25.13	15.255		24	5.50	6.200		24	31.83	9.942	
CONVID Hospitalitation	135	19.55	11.319		135	9.15	2.393		135	11.64	2.436		135	24.46	13.291		135	4.96	5.133		135	32.20	9.151	
Triage	64	19.86	10.190		64	8.86	2.21		64	11.67	3.045		64	26.70	12.693		64	4.75	5.657		64	33.36	9.072	
ICU	96	19.92	11.514		96	8.49	2.555		96	10.99	2.812		96	23.63	13.464		96	4.41	5.111		96	30.66	10.538	
COVID Primary Health Care	68	22.75	11.417		68	9.59	2.564		68	11.35	2.567		68	24.10	13.810		68	4.43	6.102		68	32.76	8.779	
Others	78	21.06	10.283		78	9.15	2.285		78	11.51	2.173		78	26.64	13.966		78	4.40	5.028		78	33.63	8.923	
Total	782	20.04	10.719		782	8.98	2.39		782	11.45	2.516		782	24.75	13.333		782	4.84	5.394		782	31.80	9.498	
Professional Experience																								
0 a 12 months	95	24.66	11.394	0.002	95	8.99	2.636	0.485	95	10.75	2.419	0.001	95	24.54	13.593	0.193	95	6.64	5.82	0,000	95	29.13	10.4	0.029
1 - 3 years	122	19.48	10.960		122	8.87	2.787		122	10.95	2.987		122	22.27	14.782		122	6.06	6.311		122	30.73	11.070	
4 - 6 years	127	19.32	10.845		127	9.08	2.195		127	11.63	2.350		127	26.47	12.135		127	5.09	5.577		127	32.99	8.695	
7 - 8 years	80	19.69	11.761		80	8.68	2.759		80	11.24	2.616		80	25.28	14.273		80	4.70	4.737		80	30.68	10.896	
9 - 10 years	82	21.35	10.145		82	9.28	2.306		82	11.94	2.168		82	24.48	11.977		82	4.91	5.329		82	32.43	8.798	
10 - 15 years	134	18.92	9.451		134	9.29	2.070		134	12.13	2.147		134	24.25	13.133		134	3.97	4.532		134	32.96	7.518	
16 - 20 years	52	18.04	10.269		52	8.60	2.207		52	11.56	2.682		52	24.98	14.022		52	3.33	4.480		52	33.79	8.788	
21 - 30 years	69	18.48	10.019		69	8.74	2.187		69	11.16	2.660		69	27.68	12.092		69	3.33	5.150		69	32.22	9.310	
More than 30 years	21	20.1	9.985		21	8.81	1.470		21	11.57	1.805		21	21.71	13.900		21	2.52	3.027		21	31.10	8.372	
Total	782	20.04	10.719		782	8.98	2.390		782	11.45	2.516		782	24.75	13.333		782	4.84	5.394		782	31.80	9.498	
AGE (in years)																								
18 - 28	162	22.20	11.799	0.063	162	8.87	2.864	0.386	162	10.83	3.006	0.001	162	22.09	14.086	0.036	162	6.01	6.015	0.000	162	28.61	11.446	0.000
29 - 38	309	19.90	10.471		309	9.17	2.396		309	11.59	2.264		309	25.42	12.754		309	5.21	5.589		309	31.79	8.941	
39 - 48	187	19.40	10.447		187	8.98	2.096		187	11.76	2.469		187	26.24	12.963		187	4.14	4.348		187	33.64	8.194	
49 - 58	108	18.23	9.799		108	8.66	2.065		108	11.19	2.405		108	23.73	13.564		108	3.15	4.728		108	32.92	9.230	
59 - 66	15	20.67	11.242		15	8.60	2.230		15	13.07	1.280		15	28.80	16.328		15	5.73	6.943		15	34.93	6.158	
More than 66	1	18.00	´----		1	11.00	´----		1	13.00	´----		1	18.00	´----		1	0.00	´----		1	39.00	´----	
Total	782	20.04	10.719		782	8.98	2.390		782	11.45	2.516		782	24.75	13.333		782	4.84	5.394		782	31.80	9.498	
SHIFT																								
Fixed morning	18	17.67	12.219	0.067	18	8.83	2.229	0.922	18	11.50	3.808	0.193	18	25.28	13.145	0.260	18	4.56	4.062	0.684	18	26.00	12.811	0.010
Fixed afternoon	1	27.00	´----		1	10.00	´----		1	12.00	´----		1	38.00	´----		1	2.00	´----		1	37.00	´----	
Fixed night	3	24.33	6.658		3	7.67	0.577		3	13.67	4.163		3	18.33	18.824		3	5.00	4.359		3	38.00	1.000	
8 hours	199	19.56	10.339		199	9.00	2.340		199	11.49	2.528		199	24.06	12.684		199	4.30	5.618		199	32.20	9.647	
12 hours	475	19.71	10.499		475	8.96	2.418		475	11.32	2.461		475	24.50	13.544		475	5.03	5.307		475	31.37	9.503	
24 hours	86	23.23	12.131		86	9.10	2.459		86	11.97	2.379		86	27.66	13.384		86	5.09	5.656		86	34.20	7.726	
Total	782	20.04	10.719		782	8.98	2.390		782	11.45	2.516		782	24.75	13.333		782	4.84	5.394		782	31.80	9.498	
Incremento horas de trabajo																								
No	485	19.13	10.299	0.005	485	8.73	2.369	0,000	485	11.38	2.546	0.484	485	22.84	13.022	0,000	485	4.47	5.214	0.042	485	31.45	9.651	0.386
Yes, to cover leave due to COVID-19	129	20.7	10.731		129	9.11	2.566		129	11.44	2.528		129	27.38	12.672		129	5.64	5.958		129	32.61	9.894	
Yes, due to lack of personnel	168	22.14	11.597		168	9.60	2.197		168	11.65	2.423		168	28.24	13.735		168	5.29	5.384		168	32.2	8.715	
Total	782	20.04	10.719		782	8.98	2.390		782	11.45	2.516		782	24.75	13.333		782	4.84	5.394		782	31.8	9.498	

Contact with infected patients was identified as among the predominant factors related to care during the COVID-19 pandemic, and majority of the participants (*n* = 685, 87.59%) had contact with COVID-19 patients daily and at least once a week. The rotation of services as a measure of reorganisation of care services accounted for 18.2% (*n* = 142), and 13.7% (*n* = 107) of participants extended their working shifts. A significant 88.88% (*n* = 695) and 21.5% (*n* = 168) of the participants respectively reduced and suspended their leave due to lack of staff, while 16.5% (*n* = 129) could not take a leave due to staff leaving because of COVID infection. Regarding job change, 5.9% of the participants (*n* = 45) switched jobs from a private to a public institution as they sought better work situations (e.g. permanent appointment). More than half (*n* = 398, 50.9%) had experience of about 10 months in the care of COVID-19 patients.

Many participants (*n* = 331, 42.33%) were diagnosed with COVID-19, although 24.8% (*n* = 194) did not undergo PCR examination during the study period. The time period of isolation was heterogeneous; however, the highest percentage of participants, i.e., 29.6% (*n* = 232), had to isolate for 1–20 days, and 18.8% (*n* = 147) represented the group of professionals who had to leave home for fear of infecting their family members and co-workers.

Regarding the repercussions of being infected with COVID-19, 25.44% of the participants (*n* = 199) reported post-COVID sequelae, the most frequent conditions being muscle involvement, pulmonary and cardiac involvement, headache, hair loss and, less frequently, decreased vision, and loss of taste and smell.

Among the factors related to the management and provision of resources and COVID-19 prevention and mitigation measures, 39.5% of the participants (*n* = 309) considered that there was inadequate protection of professionals due to difficulties related to the provision of personal protective equipment, availability of drugs and other supplies, materials and equipment, a situation that worsened at the peak of the pandemic.

During the study period, 71.61% of the participants (*n* = 560) had been vaccinated with the third dose of Pfizer, while 15% had been vaccinated with AstraZeneca. Among the reasons for preference for the Pfizer vaccine, 45% (*n* = 354) referred to the review of scientific evidence, while 31% (n = 246) mentioned secondary events with other vaccines as the main reason.

The most frequent secondary events reported are general malaise, local reactions (pain and heat) at the puncture site, and systemic reactions such as headache, fever, and chills. The perception regarding the vaccine was another factor analysed in the study, with 91% of the participants (*n* = 744) referring to the importance of the vaccine in reducing severe symptoms and likelihood of death from COVID-19. However, 38% (n= 298) expressed mistrust in vaccine administration due to secondary reactions, and 15% of respondents (*n* = 118) were sceptical about the efficacy of the vaccine.

**Fig. 1:**
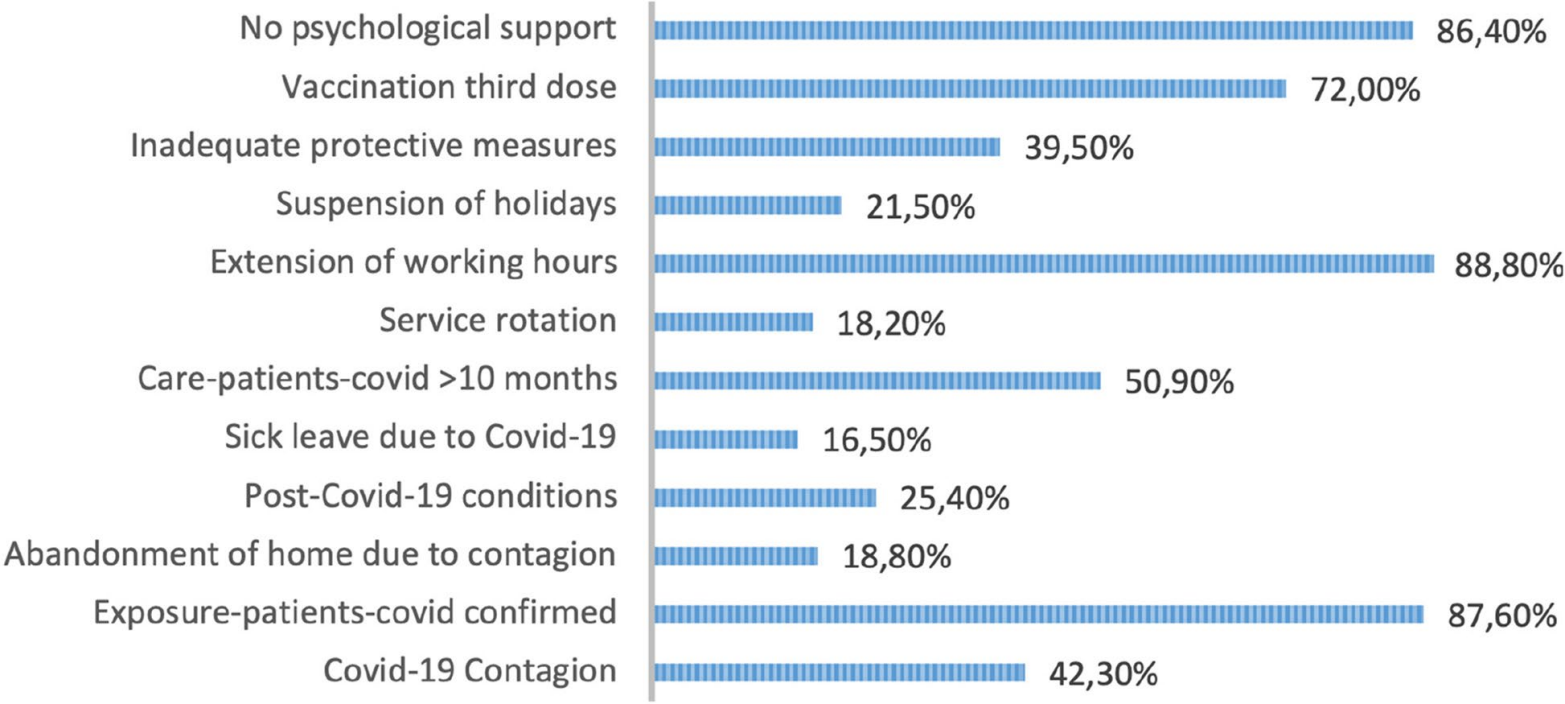
Factors related to the COVID-19 pandemic for emotional disorders among nursing professionals

Regarding the psychological support provided by health establishments, at the end of the data collection phase, 86.4% (n= 676) did not receive any type of psychological support, and 49.4% (*n* = 386) expressed the need to receive it (Figure 1).

### Mental health of nursing professionals

#### Emotional burnout

Most professionals who presented high levels of emotional exhaustion were female (*n* = 181, 90%). Emotional exhaustion was mostly observed in those aged 29–38 years (*n* = 75, 37.3%), who worked in hospitalisation services (*n* = 141, 70%), and with higher proportions who worked in intensive care units (19.9%; *n* = 156) and triage (18.86%; *n* = 147). Among the participants who admitted to experiencing burnout, more than half (*n* = 114, 56%) experienced burnout after working long shifts of 12 h,, while many others (54.7%; *n* = 428) felt it after contact with COVID-19 patients at least once a week, and some others (*n* = 58, 28.9%) experienced it after managing patients for 0–2 months.

No significant relationships were found between emotional burnout and age, gender, experience, and type of health service. However, significant values for the presence of emotional burnout were found among participants who worked night shifts (p < 0.039). Furthermore, a lower risk of burnout was observed among participants who worked 24-h shifts (OR = 0.483; CI: 0.299–0.781), which can be explained by the longer rest time between shifts.

#### Anxiety and depression

It was observed that nursing professionals presented a higher risk of anxiety when they worked eight-hour shifts, which is justified in the same way as burnout (OR = 1.284; CI: 1.192–1.383). Compared with male nurses, female nurses had 2.4 times the risk of anxiety (OR = 2.4; CI: 1.21–4.74) and 1.6 times the risk of depression (OR = 1.6; CI: 1.01–2.54). In participants aged 49–58 years, there was a higher risk of anxiety than in the other groups (OR = 2.01; CI: 1.04–3.87).

In depression, working time with COVID-19 patients for 5–7 months was found to present less risk of depression than shorter or longer working times, where limited experience and burnout played a major role as a cause of depression among professionals.

#### Post-traumatic stress disorder

According to the responses by participants to IES-R, it was found that women accounted for 92.3% (*n* = 60) of the total number of subjects who presented severe distress. Most of the participants who presented with the symptoms of PTSD were aged 29–38 years (*n* = 28, 43.1%), and 63.1% of them (*n* = 41) worked in hospitalisation services in contact with COVID-19 patients, with a working day of 12 h, at least one day per week. More than half of these participants (*n* = 34, 52.3%) worked more than 10 h per week caring for COVID-19 patients.

Analysis of the total IES-R score revealed that the probability did not vary depending on the sex of the professionals, nor did it depend on the participant’s age. However, it was found that professionals who remained in the care of COVID patients for more than 10 months were 2.31 times more likely to have post-traumatic stress than the others (OR = 2.313; CI: 1.255–4.265).

#### Burnout

Most participants who admitted to be suffering from burnout syndrome were female professionals (*n* = 423, 88.86%). Burnout was more common among those aged between 29 and 38 years (*n* = 194, 40.75%). Most of those suffering from burnout worked in hospitalisation services (*n* = 357, 75%), had a working day of 12 h (*n* = 182, 38.23%), or had contact with COVID patients at least one day per week (*n* = 41, 63.1%).

More than half of the participants with burnout syndrome (*n* = 268, 56.31%) have spent more than 10 months of professional experience in the care of this type of patient. There are statistically significant relationships in professionals with work experience between 5–7 months (p < 0.000).

From the burnout score, it is observed that the risk of burnout was independent of age and sex in the professionals studied. Also, the duration of the shifts in which the professionals interacted with COVID-19 patients did not denote a risk for burnout. In regard to the exposure time in the care of COVID patients, it was shown that the longer the working hours, the greater the probability of presenting burnout (OR = 2.28; CI: 1.267–4.113).

### Predictive regression models and correlations between scales

The results obtained in the binary logistic regression model when applying the goodness of fit test showed that *p* > 0.005, so the null hypothesis that the age of the professionals does not imply a risk of presenting anxiety, depression, or post-traumatic stress is accepted, in contrast to presenting exhaustion and burnout, where the alternative hypothesis is accepted, according to which age is considered a predisposing factor for these symptoms (p < 0.005), demonstrating a good adjustment of the test (Table 3).

**Table 3:** Binary regression

**Dependent Variable**	**Predictor Variable**	**B**	**SE**	**Exp (β)**	**Wald/t**	**P**	**R** ^**2**^ ** de Cox y Snell**	**R** ^**2**^ ** de Nagelkerke**
**Age**	Constant							
	Exhaustion	− 0.644	0.217	0.525	8.785	0.003		
	Anxiety	− 0.282	0.18	0.754	2.341	0.126		
	Depression	0.085	0.167	1.089	0.261	0.609		
	Post-traumatic stress	0.063	0.155	1.065	0.163	0.686		
	Burnout	0.43	0.154	1.538	7.769	0.005		
**Gender**	Exhaustion	− 0.042	0.3	0.959	0.02	0.887	0.017	0.026
	Anxiety	0.97	0.351	2.638	7.633	0.006		
	Depression	0.399	0.239	1.491	2.795	0.095		
	Post-traumatic stress	0.014	0.236	1.015	0.004	0.951		
	Burnout	− 0.085	0.239	0.919	0.126	0.723		
**Experience in COVID-19 Units**	Depersonalisation	0.046	0.201	1.047	0.053	0.819		
	Anxiety	0.27	0.18	1.31	2.265	0.132	0.014	0.022
	Depression	− 0.362	0.164	0.696	4.89	0.027		
	Post-traumatic stress	0.069	0.156	1.072	0.197	0.657		
	Burnout	− 0.315	0.158	0.729	3.971	0.046		

Statistical model: Cox and Snell R

As for gender, it was shown that anxiety is not related to this variable, accepting the null hypothesis, in this sense it is questionable whether gender is a risk factor. The significant relationship between gender and the probability of presenting depression is accepted.

In the case of the length of time that the professionals worked in services with COVID-19 patients, it was shown that only depression maintains its relationship with this variable, accepting the alternative hypothesis that states that a moderate length of time between 5 and 7 months allows the professional not to present with the symptoms of depression, which could appear in shorter or longer periods of exposure.

The aforementioned results make it easier to understand that the applied model presents a good fit.

### Discussion

The study found that workload and inpatient care, contact with COVID-19 patients, and less experience in disease management may be influencing factors for the presence of emotional exhaustion in nurses. A study of Mexican nurses showed a high level of emotional exhaustion, where 45.24% of nurses showed moderate-to-severe psychological distress. In addition, female nurses exhibited significantly greater psychological distress compared with male nurses. The response to COVID-19-related traumatic distress was measured using the IES-R scale, showing that 46.75% of respondents had a moderate to severe distress response, 30.95% had a mild response, and 22.30% had a normal response. The MBI-EE subscale, used to measure emotional exhaustion, indicated that 30.30% of nursing staff had a high level, 34.20% had a medium level, and 35.49% had a low level of emotional exhaustion. According to the K10 scale, 45.24% presented moderate to severe psychological distress, 14.06% had mild distress, and 40.70% had normal psychological discomfort. Female nurses showed greater psychological disturbances compared to male nurses. [35].

In regard to the characteristics of the population studied, it was observed that female professionals exhibited higher levels (score > 20) of both anxiety and depression. In contrast, male professionals were more likely to present with depressive symptoms, with higher levels than those found in an Iranian study, in which 60.2% of nursing staff presented with anxiety symptoms [36] and other studies in the context of the pandemic in China [37] and Peru [38].

The results of our study are similar to the data provided by the study of Cecere, Novellis and Gravente in Italy, with 140 critical care nurses affirming that personal accomplishment and the total DASS score have a direct relationship on quality of work-life [OR = 0.21; 95% CI (0.05–0.82); *p* = 0.024 and OR = 4.18; 95% CI (1.01–17.33); *p* = 0.049, respectively] [39].

Severe post-traumatic stress was more significant among professionals with long working hours and more likely in male professionals and with daily exposure to COVID patients. This observation contrasted with the study by Molina-Mula et al. [18],which found moderate levels of post-traumatic stress among general nurses as well as mild levels of post-traumatic stress in 37% and moderate levels in 39% of the participants. These figures are higher than those reported in China, where 16% of nurses exposed to COVID-19 were reported to have experienced post-traumatic stress [40].

The most representative percentages of burnout were found in professionals assigned to nursing care services who worked in COVID-19 hospitalisation services in second-level care hospitals. Young professionals with less work experience and with fixed night shifts are seriously affected. The conditions analysed have also been reported in the study conducted in Brazil by Silva and Silva [41].

As burnout syndrome is a direct consequence of professional factors [42] (we observed a significant level of emotional exhaustion among nurses working in COVID-19 hospital wards and ICUs, with respondents scoring more than 31 points in this dimension of burnout. A score of 26 points, the threshold indicating risky levels of emotional exhaustion [43], was reached after two months of exposure to COVID-19 units, and scores continued to rise with increased months of exposure.

Additionally, we noted higher levels of emotional exhaustion among nurses with less experience. Although elevated levels of emotional exhaustion were already present among nurses before the COVID-19 pandemic, our findings showed that nurses in COVID-19 units and ICUs were more times likely to experience emotional exhaustion [44]. Prior to the pandemic, the prevalence of high levels of emotional exhaustion among nurses ranged from 23 to 30%, with the highest observed score being much points more. A systematic review and meta-analysis conducted just before the pandemic, which explored burnout among nurses, confirmed that younger age and less experience were associated with burnout, along with factors related to workload and work environment. This systematic review reported findings similar to ours, indicating a prevalence of 34% for high levels of emotional exhaustion [14].

On the other hand, it was found that the workload can be a key factor in the affectation of psycho-emotional disorders among nursing professionals, the main reasons reported being the increase in the working day, the suspension of days off and holidays, and the lack of personnel to attend to the demand of patients. The prevalence rate found in our study contrasts with the findings of a study carried out in Spain, where the change of usual shift is significantly associated with depression, professional profile with anxiety, and perceived stress with age [45].

Shift work (many of these nocturnal), poor recognition from patients, continuous interaction with patients and their families, which sometimes generates a constant demand, and continuous exposure to pain or death are presented as significant stressors in the profession [46].

Shift work generates a plethora of negative consequences for professionals. Over the years, a wide variety of studies have shown that these consequences can manifest both physically and psychologically. Among the repercussions that can be observed are disruptions to the circadian rhythm and sleep disturbances, as well as the disruptions to social and familial life due to the limitations imposed by shift work [47].

According to the study by Arias F et al., Bestratén in 2000 demonstrated that “shift work entails a contradiction between the various social synchronizers and the organism, resulting in what is known as ‘shift work pathology,’ characterized by asthenia, nervousness, and dyspepsia” [48].

Shift work has negative consequences on the quality of life of workers [8]. Research results show that night shift workers experience greater emotional exhaustion, while morning shift workers experience more depersonalization and dissatisfaction [49].

All the factors inherent to work reveal the adaptations that have occurred in health services, becoming stressors and potential aggravators of psychological disorders [50]. Thus, for example, deficits in medical supplies and protective material would generate a greater presence of psycho-emotional disorders. These results show similarities with other studies in which the main factors of dissatisfaction with the management of the pandemic have been found to be the lack of resources, availability of medicines, lack of personal protective equipment [51], as well as training and work induction processes [52]. In addition, there is evidence of a health crisis in Ecuador, related to other factors associated with the management of mitigation and control measures at the beginning of the pandemic.

Some elements act as predisposing factors to stress, burnout, anxiety, and depression among nursing professionals who faced the COVID-19 pandemic, such as fear of contagion, uncertainty about the effectiveness of vaccines, presence of comorbidities, safety in the protocols for carrying out diagnostic tests, and the after-effects in the case of infected professionals [53].

### Limitations

This study has a few limitations, which are addressed herein. The study sample consisted of nurses from the health establishments of the health institutions providing health services with the greatest coverage of Zone 1 of Ecuador.

We consider that establishing a series of ranges in some variables such as age and professional experience has posed a limitation for analyzing these variables as quantitative. we must state that survival analysis (Cox Regression) was not conducted because time as a ratio variable is imperative for performing Cox and Shell analyses, and it was not recorded in the database. As an alternative, we attempted to use nurses' age variable as the time variable, but it is not specified as a ratio variable (scalar), rather as an interval variable (ages are in ranges).

However, the fact that a representative sample was obtained may differ from other samples, including other institutions that are components of the < BLINDED FOR REVIEW > , in the control of the COVID-19 pandemic. There may be response bias owing to the probabilities of access to the questionnaire in nurses from urban and marginal urban areas.

### Conclusion

Based on our results, we conclude that the pandemic's effects greatly impacted the emotional and mental health of nursing professionals in < BLINDED FOR REVIEW > . Socio-demographic and occupational variables were identified that significantly predicted the probability of presenting anxiety, depression, and post-traumatic stress disorders.

Concerns about COVID-19 and perceptions about the measures implemented during the pandemic are factors that influenced the resilience of professionals in the context of the pandemic.

This study provides recommendations to the managers of health facilities in < BLINDED FOR REVIEW > for implementing measures and strategies to improve mental health in the workplace, the control of stressors, and specialised intervention in cases of risk. The results of this study have highlighted the role and challenges of nursing professionals in our country during the pandemic.

### Relevance for clinical practice

This study provides relevant information on the mental health of nurses after the COVID-19 pandemic and establishes different relationships between variables that allow health systems to establish strategies to improve the quality of life of these professionals.

Knowing which characteristics, environments, and processes are most damaging to nurses is critical to proactively address their emotional needs and prevent a breakdown of the healthcare system. Moreover, as the study has shown, accounting for the environments where the emotional impact is greatest and how to reduce it would not only reduce anxiety, depression, and burnout in nurses but also improve the quality of care, not only in pandemic situations but also in the future.

### Supplementary Information

Supplementary Material 1.

## Data Availability

The complete analysis data and results matrix can be requested through the corresponding author's email.

## References

[1] Nwanonyiri DC, Ogiehor-Enoma G, Iwu E, Nwaneri T. Helping nurses cope with COVID-19 pandemic: evaluating supports programs. Open J Nurs. 2021;11(2):65–74.

[2] Moreno-Casbas MT. Factores relacionados con el contagio por SARS-CoV-2 en profesionales de la salud en España. Proyecto SANICOVI Enferm Clin. 2020;30(6):360–70.10.1016/j.enfcli.2020.05.021PMC724750432571661

[3] García Morán MD, Cruz M. El estres en el ámbito de los profesionales de la salud. Persona. 2016;19:11–30.

[4] Luo M, Guo L, Yu M, Wang H. The psychological and mental impact of coronavirus disease 2019 (COVID-19) on medical staff and general public – a systematic review and meta-analysis. Psychiatry Res. 2020;291:113190. 10.1016/j.psychres.2020.113190 PMC7276119 32563745

[5] Montes-Berges B, Fernández-García E. El efecto de la pandemia en la salud y Síndrome de Burnout en profesionales de enfermería de UCI. Enferm Glob. 2022;21(2):15–27.

[6] El-Hage W, Hingray C, Lemogne C, Yrondi A, Brunault P, Bienvenu T, et al. Health professionals facing the coronavirus disease 2019 (COVID 19) pandemic: what are the mental health risk? Encephale. 2020;46(3):73–80. 10.1016/j.encep.2020.04.008 PMC7174182 32370984

[7] Allen JP, Crawford EF, Kudler H. Nature and treatment of comorbid alcohol problems and post traumatic stress disorder among american military personnel and veterans. Alcohol Res. 2016;38(1):133–40. 10.35946/arcr.v38.1.16 PMC4872608 27159820

[8] Emergencia Nacional C. Informe de situación COVID-19 Ecuador. Servicio Nacional de Gestión de riesgos y emergencias. Quito: Ecuador Totales. 2021.

[9] Serafini G, Parmigiani B, Amerio A, Aguglia A, Sher L, Amore M. The psychological impact of COVID-19 on the mental health in the general population. QJM. 2020;113(8):531–7. 10.1093/qjmed/hcaa201 PMC7337855 32569360

[10] Luceño-Moreno L, Talavera-Velasco B, García-Albuerne Y, Martín-García J. Symptoms of posttraumatic stress, anxiety, depression, levels of resilience and burnout in spanish health personnel during the COVID-19 pandemic. Int J Environ Res Public Health. 2020;17(15):5514. 10.3390/ijerph17155514 PMC7432016 32751624

[11] Nowicki GJ, Ślusarska B, Tucholska K, Naylor K, Chrzan-Rodak A, Niedorys B. The severity of traumatic stress associated with covid-19 pandemic, perception of support, sense of security, and sense of meaning in life among nurses: research protocol and preliminary results from Poland. Int J Environ Res Public Health. 2020;17(18):1–18. 10.3390/ijerph17186491 PMC7559728 32906590

[12] Zakeri MA, Hossini Rafsanjanipoor SM, Zakeri M, Dehghan M. The relationship between frontline nurses’ psychosocial status, satisfaction with life and resilience during the prevalence of COVID‐19 disease. Nurs Open. 2021;8(4):1829–39. 10.1002/nop2.832 PMC8186693 33675182

[13] Fornés-Vives J, García-Banda G, Frias-Navarro D, Pascual-Soler M (2018). Longitudinal study predicting burnout in Spanish nurses: The role of neuroticism and emotional coping. Pers Individ Dif.

[14] Hill J, Harris C, Danielle L, Boland P, Doherty A, Benedetto V, et al. The prevalence of mental health conditions in healthcare workers during and after a pandemic: Systematic review and meta-analysis. J Adv Nurs. 2022;78(6):1551–73. 10.1111/jan.15175 PMC9111784 35150151

[15] Planchuelo A, Odriozola P, Irurtia M, Luis R. Longitudinal evaluation of the psychological impact of the COVID-19 crisis in Spain. J Affect Disord. 2020;277:842–9. 10.1016/j.jad.2020.09.018 PMC7476580 33065825

[16] Sánchez JP, González T, Pool S, López M, Tovilla C. Estado emocional y psicológico del personal de enfermería agredido durante la pandemia de COVID-19 en Latinoamérica. Rev Colomb Psiquiatr. Spanish. 2021. 10.1016/j.rcp.2021.08.006. 10.1016/j.rcp.2021.08.006 PMC8498689 34642505

[17] Lahav Y. Psychological distress related to COVID-19 – The contribution of continuous traumatic stress. J Affect Disord. 2020;277:129–37. 10.1016/j.jad.2020.07.141 10.1016/j.jad.2020.07.141 PMC7416772 32818776

[18] Molina-Mula J, González A, Perelló C, Tera J, Otero L, Romero N. The emotional impact of COVID-19 on Spanish nurses and potential strategies to reduce it. Collegian. 2022;29(3):296–310. 10.1016/j.colegn.2021.12.004. 10.1016/j.colegn.2021.12.004 PMC8666309 34924803

[19] García-Iglesias JJ, Gómez J, Martín J, Fagundo J, Ayuso D, Martínez J, et al. Impacto del SARS-CoV-2 (Covid-19) en la salud mental de los profesionales sanitarios: una revisión sistemática. Rev Esp Salud Publica. 2020;94(1):1–20. PMC11582971 32699204

[20] Mathieu E, Ritchie H, Rodés L, Appel C, Giattino C, Hasell J, et al. Our World. Coronavirus Pandemic (COVID-19). 2020. Published online at OurWorldInData.org. Retrieved from: https://ourworldindata.org/coronavirus.

[21] Mena A, Casali P. Organización Internacional del Trabajo OIT. In: Obtenido de El sistema de salud ecuatoriano y la COVID-19. Ecuador. 2020.

[22] Vinueza A, Aldaz N, Mera C, Tapia E, Vinueza M. Síndrome de Burnout en personal sanitario ecuatoriano durante la pandemia de la COVID-19. Correo Científico Médico. 2021;25(2):1–17.

[23] Pappa S, Ntella V, Giannakas T, Papoutsi E, Katsaounou P. Corrigendum to “Prevalence of depression, anxiety, and insomnia among healthcare workers during the COVID-19 pandemic: A systematic review and meta-analysis. Brain Behav Immun. 2020;88:901–7. 10.1016/j.bbi.2020.05.026. 10.1016/j.bbi.2020.05.026 PMC7206431 32437915

[24] Instituto Nacional de Estadística y Censos. Registro Estadístico de Recursos y Actividades de Salud 2006-2018. Instituto Ecuatoriano de Estadísticas y Censos. Ecuador. 2020.

[25] Oceano Medicina. Oceano Medicina. 2022. Available from: https://ec.oceanomedicina.com/nota/enfermeria/ecuador-todavia-no-alcanza-el-numerode-profesionales-de-enfermeria-recomendado-por-la-oms/.

[26] Pita S, González C, Pillado S, López B, Pértega S, Gil V. Validity of footprint analysis to determine flatfoot using clinical diagnosis as the gold standard in a random sample aged 40 years and older. J Epidemiol. 2015;25(2):148–54. 10.2188/jea.JE20140082. 10.2188/jea.JE20140082 PMC4310876 25382154

[27] Gil P, Peiro J. Validez factorial del maslach burnout inventory en una muestra multiocupacional. Psicothema. 1998;11(3):679–89.

[28] Terol M, Cabrera V, Martín M. Revisión de estudios de la Escala de Ansiedad y Depresión Hospitalaria (HAD) en muestras españolas. Anal. Psicol. 2015;31(2):494–503. 10.6018/analesps.

[29] López S, Terol M, Pastor M, Neipp M, Massuti B, Rodríguez M. Ansiedad y depresión. Validación de la escala HAD en pacientes oncológicos. Rev Psicol Salud. 2000;12(2):127–51.

[30] Costa G, Gil F. Propiedades psicométricas de la escala revisada del impacto del evento estresante (IES-R) en una muestra española de pacientes con cáncer. Anal Modif Cond. 2007;33(149):11–331.

[31] Torres Toala G, Irigoyen Piñeiros V, Paola Moreno A, Ruilova Coronel EA, Casares Tamayo J, Mendoza Mallea M. Burnout syndrome in health professionals in Ecuador and associated factors in times of pandemic. Rev virtual Soc Parag Med Int. 2021;8(1):1–11.

[32] Breilh J. Trabajo hospitalario, estrés y sufrimiento mental: deterioro de la salud de los internos en Quito, Ecuador. Ateneo; 2009. p. 8–19.

[33] Gualpa-Jaramillo GG, Choca-Alcoser EG, Basantes-Moscoso DR (2020). Trastorno de estrés Postraumatico; experiencias en Ecuador en la población de Riobamba. Pol Con.

[34] Ramírez Enríquez V, Ramos NI (2021). Estudio comparativo de niveles de ansiedad generados por el COVID- 19 en pacientes con diagnóstico previo de reacción al estrés. Ciencia Unemi.

[35] Cortés N, Vuelvas C. Estudio comparativo de los efectos psicológicos entre enfermeros y enfermeras en México durante la pandemia de la COVID-19. Naturaleza y Tecnología. 2022;1:21–35.

[36] Nemati M, Ebrahimi B, Nemati F. Assessment of Iranian Nurses’ knowledge and anxiety toward COVID-19 during the current outbreak in Iran. Arch Clin Infect Dis. 2020;15(COVID-19):e102848.

[37] Guo J, Liao L, Wang B, Guo L, Tong Z, Guan Q, et al. Psychological effects of COVID-19 on hospital staff: a national cross-sectional survey of China Mainland. Harv Public Health Rev (Camb). 2020;28. http://harvardpublichealthreview.org/wp-content/uploads/2020/10/Deng-and-Naslund-2020-28.pdf.

[38] Yslado R, Nuñez L, Montane L, Bobadilla R, Cruz L, Pinto I, et al. Síndrome de Burnout, ansiedad, depresión y bienestar laboral en personal hospitalario de Perú durante la pandemia de COVID-19. Rev Cuba Invest Bioméd. 2022;41:e2555.

[39] Cecere L, De Novellis S, Gravante A, Petrillo G, Pisani L, Terrenato I, et al. Quality of life of critical care nurses and impact on anxiety, depression, stress, burnout and sleep quality: A cross-sectional study. Intensive Crit Care Nurs. 2023;79:103494. 10.1016/j.iccn.2023.103494j.iccn.2023.103494. Cited 2024 May 29. 10.1016/j.iccn.2023.103494 37556987

[40] González A, Labad J. Salud mental en tiempos de la COVID: reflexiones tras el estado de alarma. Med Clin (Barc). 2020;155(9):392–4. 10.1016/j.medcli.2020.07.009. 10.1016/j.medcli.2020.07.009 PMC7381887 32958264

[41] Silva R, Silva V. Pandemia de la COVID-19: síndrome de Burnout en profesionales sanitarios que trabajan en hospitales de campaña en Brasil. Enferm Clin. 2021;31(2):128–29. 10.1016/j.enfcli.2020.10.011. 10.1016/j.enfcli.2020.10.011 PMC7603962 38620439

[42] Maslach C, Jackson SE, Leiter MP, Schaufeli WB, Schwab RL. Maslach burnout inventory. vol. 21. 3rd ed. Palo Alto: Consulting psychologists press; 1997.

[43] Maslach C, Jackson SE. The measurement of experienced burnout. J Organ Behav. 1985;2(2):99–113.

[44] Gómez-Urquiza JL, Monsalve-Reyes CS, San Luis-Costas C, Fernández-Castillo R, Aguayo-Estremera R, Fuente GA. Factores de riesgo y niveles de burnout en enfermeras de atención primaria: una revisión sistemática. Aten Primaria. 2017;49(2):77–85. 10.1016/j.aprim.2016.05.004. 10.1016/j.aprim.2016.05.004 PMC6876264 27363394

[45] Carranza S, Mamani O, Quinteros D, Farfán R. Preocupación por el contagio de la COVID-19 y carga laboral como predictores del malestar psicológico durante la emergencia sanitaria en personal de salud de Perú. Rev Colomb Psiquiatr (Engl Ed). 2021. 10.1016/j.rcp.2021.06.005. 10.1016/j.rcp.2021.06.005 PMC8249714 34230700

[46] Contreras F, Juárez F, Murrain E. Influencia del Burnout, la calidad de vida y los factores socioeconómicos en las estrategias de afrontamiento utilizadas por los profesionales y auxiliares de enfermería. Pensam Psicol. 2008;4(1):31–40.

[47] M QA, FJ LE. Evaluación del estrés laboral y burnout en los servicios de urgencia extrahospitalaria. Int J Clin Health Psychol. 2007;7(2):323–35.

[48] Arias F, Barboza N, Blanco J, Fajardo E, Rivera R, S R. Síndrome del desgaste profesional o de burnout en el personal de enfermería. 2007-2008;13:3–9.

[49] J PP. Efecto del burnout y la sobrecarga en la calidad de vida en el trabajo. Estud Gerenc. 2013;29(129):446–53.

[50] de Pontes Santos HB, Miguel MC da C, Pinto LP, Gordón-Núñez MA, Alves PM, Nonaka CFW. Multinucleated giant cell reaction in lower lip squamous cell carcinoma: a clinical, morphological and immunohistochemical study. J Oral Pathol Med. 2017;46(9):773–9. 10.1111/jop.12565 28199754

[51] Flores Romero, E. COVID-19 y comunicación de salud: Análisis de medios digitales ecuatorianos. Revista Scientific. 2021;6(19):122–41. 10.29394/Scientific.issn.2542-2987.2021.6.19.6.122-141.

[52] Trujillo-Hernández PE, Gómez-Melasio DA, Lara-Reyes BJ, Medina-Fernández IA, Hernández-Martínez EA. Asociación entre características sociodemográficas, síntomas depresivos, estrés y ansiedad en tiempos de la COVID-19. Enferm Glob. 2021;20(4):1–25.

[53] Mesa Castro N. Influencia De La Inteligencia Emocional Percibida En La Ansiedad Y El Estrés Laboral De Enfermería. ENE. 2019;3(3):1–26.

